# Evaluation of 3D printed nano-modified resin shear bond strength on titanium surfaces (an in-vitro study)

**DOI:** 10.1186/s12903-025-06223-8

**Published:** 2025-05-27

**Authors:** Noha Sabry ElMalah, Yomna Ibrahim, Dawlat Mostafa

**Affiliations:** 1 Dental Biomaterials, College of Dentistry, The Arab Academy for Science and Technology and Maritime Transport (AASTMT), El-Alamein, Egypt; 2 Dental Biomaterials Department, Faculty of Dentistry, Dental Biomaterials, Alexandria University, Alexandria, Egypt

**Keywords:** Nanoparticles, 3D printing, Nanocomposite resin, Titanium, Aging, Shear bond strength

## Abstract

**Background:**

Interim restorations are crucial in dental implant procedures as they ensure patient’s comfort, maintain esthetic appearance, and restore function during the healing process. Optimizing retention of these restorations ensures their long-term success. This study aims to evaluate the shear bond strength (SBS) of nano-modified, additively manufactured resin-based interim materials to smooth and rough titanium surfaces.

**Methods:**

Ninety-six specimens were prepared with a 3D printed resin (VarseoSmile Crown plus; Bego) and divided into 3 groups: group I (VS control) (*n* = 32), group II (VS 0.2%TiO_2_) (*n* = 32), and group III (VS 0.4%TiO_2_) (*n* = 32), then each group was divided into 2 subgroups according to bonded titanium surface: smooth (*n* = 16) and sandblasted (*n* = 16). The prepared resin samples underwent air abrasion followed by citric acid etching. Subsequently, surface roughness (Ra) values were measured by surface profilometer. Each specimen was bonded with a dual-cured adhesive resin cement for SBS testing using universal testing machine. Half of the specimens of each group were subjected to thermocycling (1000 cycles) then tested for SBS. Failure modes were determined using stereomicroscope. Surface roughness was compared using paired t-tests, while two-way ANOVA assessed filler type and surface treatment effects. Three-way ANOVA evaluated the impact of filler type, surface treatment, and thermocycling on SBS. Significance was set at *P* < 0.05.

**Results:**

Surface treatment showed a statistically significant increase in surface roughness of nanomodified composite specimens as well as titanium surfaces (*P* < 0.0001). The highest surface roughness was seen in group I (0.701 ± 0.113) followed by group III (0.690 ± 0.107), group II (0.653 ± 0.133) and rough titanium surface (0.548 ± 0.062). Regarding SBS values, before thermocycling, group I (8.85 ± 1.03) was the highest, followed by group III (8.29 ± 0.57) then group II (6.87 ± 0.53). After thermocycling, group III bonded to rough titanium surface showed the highest values (12.87 ± 0.77), while group II was the lowest (7.81 ± 0.94) (*P* < 0.0001).

**Conclusion:**

Surface treatment significantly enhanced surface roughness and SBS of nanomodified composites to titanium surfaces. This improvement underscores the effectiveness of nanomodification and surface treatment in optimizing the adhesive interface, which is crucial for achieving durable bonding in dental restorations.

**Supplementary Information:**

The online version contains supplementary material available at 10.1186/s12903-025-06223-8.

## Introduction

In modern dental practice, there is a growing trend towards fully digital workflows, driven by advances in technologies such as Computer-Aided Design (CAD) and Computer-Aided Manufacturing (CAM). These technological innovations have revolutionized clinical procedures and enabled the creation of novel restorative materials [1]. A notable transition is occurring from subtractive to additive manufacturing, particularly using 3D printing [2]. These have several advantages over the subtractive technique, which include improved cost-efficiency, quicker production times, enhanced customization, and a reduction in manual labor requirements [3].

The manufacturing process for implant-supported dental prostheses has also embraced fully digital workflows, utilizing CAD-CAM technology alongside an array of ceramic and composite materials to produce monolithic restorations, either chairside or in dental laboratories [4]. Early and immediate implant loading has become common to maintain aesthetics and support soft tissue healing, highlighting the growing importance of interim restorations [5].

Temporary crowns and fixed dental prostheses (FDPs) are essential for providing long-term stability and protecting teeth during complementary treatments. These restorations must meet aesthetic, biological, and mechanical requirements, including resistance to dislodging forces and functional loads. However, complications such as fractures can arise in extensive temporary restorations intended for prolonged use, as they are subjected to compressive, tensile, and shear forces [6].

One common issue is the debonding of restorations from Ti-base abutments, a frequent complication in monolithic ceramic implant-borne crowns and fixed partial dentures. Clinical studies have reported debonding within the first two years of clinical use across various crown and abutment materials [7, 8]. Most interim implant-supported restorations are screw-retained as they ease the retrievability of the restoration. However, they require an adequate inter-arch distance; their screw hole might compromise esthetics in the anterior region; they are more liable to chipping and fracture, especially when fabricated by 3D printing, due to lower fracture resistance than milled ones; and they are more expensive and tedious to fabricate compared to cement-retained restorations [9–11].

Hence, various surface treatments and bonding agents have been explored to enhance the bond strength of resin composites. Surface roughening techniques such as bur abrasion, acid etching, and air-borne particle abrasion enhance the retention of the luting cement [12–14]. Self-adhesive resin cements are popular for bonding CAD-CAM materials to Ti-bases due to their ease of use, though clinical evidence on newer universal products remains limited [4]. The use of functional monomers and adhesive primers, which are incorporated in most resin cements, is also considered an additional boost to the bond strength of composite restorations with titanium [15, 16].

Fouquet et al. and Donmez et al. discussed the use of various implant-supported resin restorations as well as diverse luting agents and surface treatments for the abutment and restoration surfaces [17, 18]. Despite extensive research on bonding between titanium abutments and restorative materials, there is still no established protocol for Ti-base cementation across different crown materials and cementation media, especially for cement-retained implant-supported interim restorations [5].

This study aims to evaluate a 3D printed photocurable resin modified with titanium dioxide nanoparticles (TiO_2_ NP) as a long-term interim implant-supported restorative material and its shear bond strength (SBS) to both smooth and sandblasted titanium specimens after its treatment with sandblasting and citric acid etching. The null hypothesis of this study states that there will be no significant difference in the shear bond strength of the nano-modified resin when bonded to smooth titanium surfaces or sandblasted ones.

## Material and methods

### Sample size estimation

**Fig. 1:**
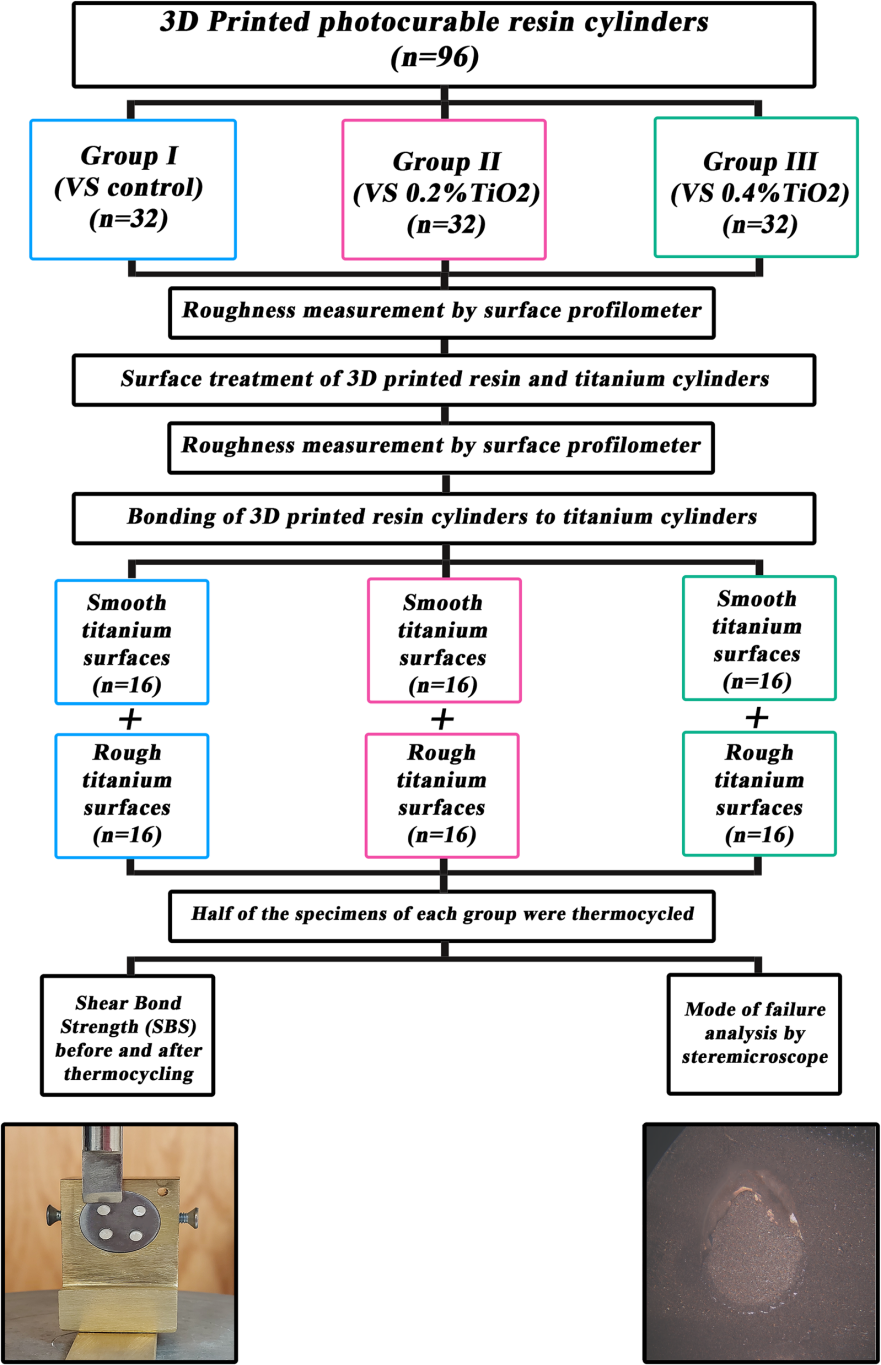
Study scheme and grouping of specimens

Sample size was based on a 95% confidence level to detect differences in shear bond strength of titanium alloy between different surface treatments. Lee et al. [15] reported mean (SD) titanium alloy shear bond strength = 19.10 (1.68) and 24.93 (1.32) after sandblasting and silicoating, respectively. The calculated mean (SD) difference = 5.83 (1.51) and the 95% confidence interval = 5.21, 7.61. The minimum required sample size = 15 per group, increased to 16 to make up for laboratory processing problems. The total required sample size = number of groups × number per group = 6 × 16 = 96 samples. Sample size was calculated using MedCalc Statistical Software version 19.0.5 (MedCalc Software bvba, Ostend, Belgium; https://www.medcalc.org; 2019). The study scheme and grouping of the specimens are shown in Fig. 1.

### Sample preparation

TiO_2_ NPs (Nanotech, Egypt) were incorporated with 3D printed dental resin (VarseoSmile Crown plus, Bego, Germany) to prepare three groups: Group I (VS control), Group II (VS 0.2%TiO_2_), and Group III (VS 0.4%TiO_2_). The mixture was sonicated for 10 min then placed under continuous magnetic stirring (F91T, FALC Instruments, Italy) for 1 h [19, 20]. Cylindrical specimens Ø3 × 3- mm were designed by a CAD software, then a Standard Tessellation Language (STL) file was loaded into a digital light processing printer (Varseo XS, Bego, Germany) [6, 17]. A printing orientation of 45° and a 50 µm layer thickness were used. Other printing parameters were set according to the manufacturer’s instructions based on the shade and type of resin used. The printed specimens were then cleaned in an ultrasonic bath (T-14, LR manufacturer, USA) using 96% isopropanol for 5 min to remove any remaining resin and then post-polymerized in a high-performance light curing unit (HiLite power 3D, Kulzer GmbH, Germany) at 200 W for 20 min [21].

### Titanium cylinders preparation

Titanium grade V rods (20 mm in diameter) were cut into cylinders of 10 mm height using a computer numerical control cutting machine (CNC) (QC400, SENYAT, China). The surface was polished then either left as it is to be used with the smooth surface group or sandblasted with 50 µm alumina particles (M&Y, China) with a blasting device (Basic eco, Renfert, Germany) to be used for the rough surface group [22].

### Surface treatment of 3D printed cylindrical specimens

The surface of all nanomodified composite specimens was subjected to air abrasion with 50 µm alumina particles with a blasting device from a 10 mm distance at 2 bar propulsion pressure for 15 s. Compressed air was then applied to clean the surface, followed by etching with 30% citric acid (Axom, India) for 60 s [23]. The surface was then cleaned by sonication in distilled water for 1 min and then dried with air spray for 30 s.

### Surface roughness and surface topography

The average roughness (Ra) for each specimen was measured using a roughness profilometer instrument (Marsurf PS10, Mahr, Germany) before and after surface treatment. Representative samples from each group were gold sputter coated, and then images were obtained at × 500 magnification and 20 kV via scanning electron microscope (SEM) (JSM-IT200, JEOL, Japan) [19].

### Bonding process

Resin cylinders were bonded to titanium cylinders using self-adhesive dual-cure resin (Nova, Imicryl, Türkiye) (fluorine containing resin cement with glycero-phosphate dimethacrylate (GPDM) and 4-META functional monomers) where a constant pressure of 20 N was applied to ensure proper adaptation [24]. The adhesive was then light cured (Woodpecker, China), with an intensity of 850 mW/cm^2^ through the specimens for 40 s. All specimens were stored in distilled water at 37 °C for 48 h prior to the SBS test [25].

### Shear bond strength test

The shear bond test was performed in accordance with the ISO 10477:2020 standard for crown material in dentistry [26]. A universal testing machine (5 ST, Tinius Olsen, England) exerted a shear force on the specimens with a crosshead speed of 0.5 mm/min from the parallel direction of the bonding surface until fracture occurred and failure load was recorded. Then SBS values of each specimen were calculated in MPa using the following equation: $=\frac{L}{A}$, where $\sigma$ is the bond strength, L is the load at failure (in N), and A is the bonded area (in mm^2^). Half of the specimens were thermocycled using a thermocycling machine (Custom-made, Egypt) applying 1,000 cycles to alternate 5 °C and 55 °C water baths with a dwell time of 30 s [12].

After SBS testing, specimens were analyzed under a stereomicroscope (B016; Olympus, Japan) to assess the mode of failure [27]. A failure mode of the following was assigned to each specimen: (I) cohesive in resin cement, (II) cohesive in 3D printed resin, (III) adhesive between resin cement and titanium surface, (IV) adhesive between resin cement and 3D printed resin, and (V) mixed failure.

### Statistical analysis

Normality was checked for all variables using descriptive statistics, plots, and normality tests. All variables showed normal distribution; thus, means and standard deviation (SD) were calculated, and parametric tests were used. Comparison of surface roughness of smooth and rough titanium specimens was done using independent paired samples t-test. Two-way ANOVA was done to assess the effect of filler type and surface treatment on surface roughness of the three study groups of VarseoSmile resin. Three-way ANOVA was performed to assess the association of filler type, surface treatment, and thermocycling on shear bond strength (SBS) of titanium and resin. Data were analyzed using IBM SPSS for Windows (Version 27.0) and significance was set at *P* value < 0.05.

## Results

There was a significant increase in the surface roughness of the titanium surfaces after sandblasting, as indicated by the t-test value (-28.4) and the *P* value of < 0.0001 (Table 1). Concerning surface roughness of nanomodified resin specimens, the addition of titanium dioxide nanoparticles did not show a statistically significant difference among study groups (*P* = 0.19) in contrast to citric acid etching and sandblasting, which showed a statistically significant increase in surface roughness (*P* < 0.0001) and their interaction was also significant at (*P* = 0.04) as shown in Table 2.

**Table 1:** Paired t-test for surface roughness of titanium surfaces

Test	t-test value	*P*
Surface roughness (µm)	-28.4	< 0.0001^*^

^*^Statistically significant difference (*P* < 0.05)

**Table 2:** Two-way ANOVA of surface roughness of 3D printed resin groups

Test	Variables	Mean square	F test	*P*	Ƞp^2^
**Surface roughness (µm)**	Filler	0.02	1.71	0.19	0.03
	Surface treatment	7.75	699.52	< 0.0001^*^	0.87
	Interaction	0.04	3.37	0.04^*^	0.06

*Ƞp*^*2*^ partial eta squared

^*^Statistically significant difference (*P* < 0.05)

**Fig. 2:**
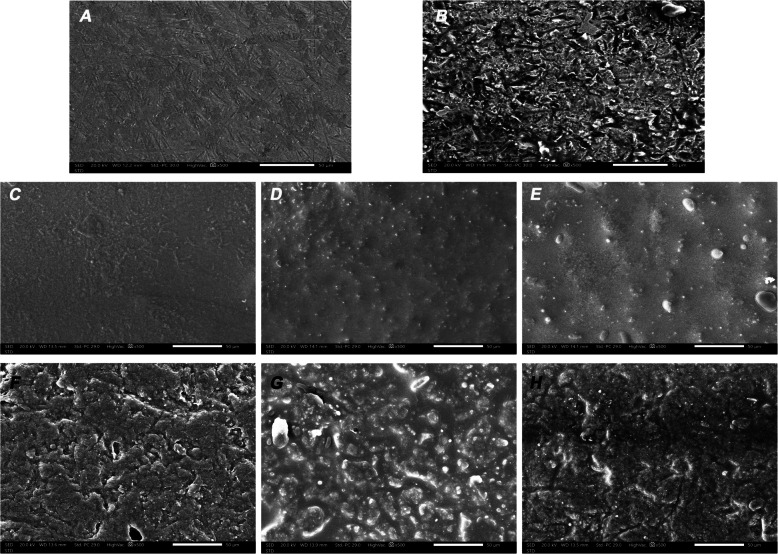
SEM images (× 500) of (**A**) smooth titanium surface, (**B**) rough titanium surface, (**C**) smooth VS control surface, (**D**) smooth VS 0.2%TiO_2_ surface, (**E**) smooth VS 0.4% TiO_2_ surface (**F**) rough VS control surface, (**G**) rough VS 0.2% TiO_2_ surface and (**H**) rough VS 0.4% TiO_2_ surface.\

SEM images (Fig. 2A, B) obtained before and after surface treatment of the titanium surface show an increase in roughness and surface irregularities after sandblasting. Figure 2C-H shows the surfaces of the 3D printed resin groups before and after surface treatment, where the surface roughness increased after surface treatment (Fig. 2F–H) compared to those before surface treatment (Fig. 2C-E). The VS control shows the smoothest surface before surface treatment (Fig. 2C) and the roughest surface after treatment (Fig. 2F) of all study groups. The groups with titanium dioxide nanoparticles showed increased roughness before surface treatment (Fig. 2D, E) compared to the control (Fig. 2C). However, after surface treatment, the titanium dioxide-filled groups showed less roughness (Fig. 2G, H) compared to the control (Fig. 2F).

The descriptive values of shear bond strength (SBS) before and after thermocycling over smooth and rough titanium surfaces for study groups are presented in Table 3. The highest mean value for SBS was found for all test groups after thermocycling, where group III showed a significant increase in SBS (12.82 ± 0.77) when compared to group I (10.21 ± 1.62) (*P* = 0.02) and group II (7.81 ± 0.94) (*P* < 0.0001) bonded to a rough titanium surface. A slight difference is seen in the SBS results before thermocycling, where group I was the highest (8.85 ± 1.03) while group II (6.87 ± 0.53) showed the least SBS value among the 3 groups with no statistically significant difference between groups I and III (Supplemental Table 1). Regarding nano modified composite specimens bonded to smooth titanium surfaces, SBS values were highest in group III after thermocycling (7.98 ± 0.89) followed by group I (7.84 ± 0.69) and group II (6.17 ± 1.54) without showing significant differences among them (Supplemental Table 1).

**Table 3:** Descriptive values of shear bond strength (SBS) before and after thermocycling over smooth and rough titanium surfaces for study groups

Titanium surface	Thermocycling	Group I (*n* = 8)	Group II (*n* = 8)	Group III (*n* = 8)
**Smooth Titanium surface**	Before thermocycling x̄ (SD)	7.77 (2.17)	5.53 (2.67)	7.27 (2.16)
	After thermocycling x̄ (SD)	7.84 (0.69)	6.17 (1.54)	7.98 (0.89)
**Rough Titanium surface**	Before thermocycling x̄ (SD)	8.85 (1.03)	6.87 (0.53)	8.29 (0.57)
	After thermocycling x̄ (SD)	10.21 (1.62)	7.81 (0.94)	12.82 (0.77)

*SD* Standard deviation, *x̄* mean

The results of the three-way ANOVA of SBS show that the three variables: filler content (Ƞp^2^ = 0.39), surface treatment (Ƞp^2^ = 0.36) and thermocycling (Ƞp^2^ = 0.20) resulted in statistically significant increase in SBS values of nanomodified composite specimens (*P* < 0.0001) (Table 4).

**Table 4:** Three-way ANOVA of shear bond strength (SBS) of 3D printed resin groups to smooth and rough titanium surfaces with and without thermocycling

Test	Variables	Mean square	F test	*P*	Ƞp^2^
**Shear bond strength (MPa)**	Filler	56.98156	26.4111	< 0.0001^*^	0.39
	Surface treatment	100.48688	46.57592	< 0.0001^*^	0.36
	Thermocycling	45.56579	21.11986	< 0.0001^*^	0.20
	Interaction	6.59269	3.05573	0.05	0.07

*Ƞp*^*2*^ partial eta squared

^*^Statistically significant difference (*P* < 0.05)

**Fig. 3:**
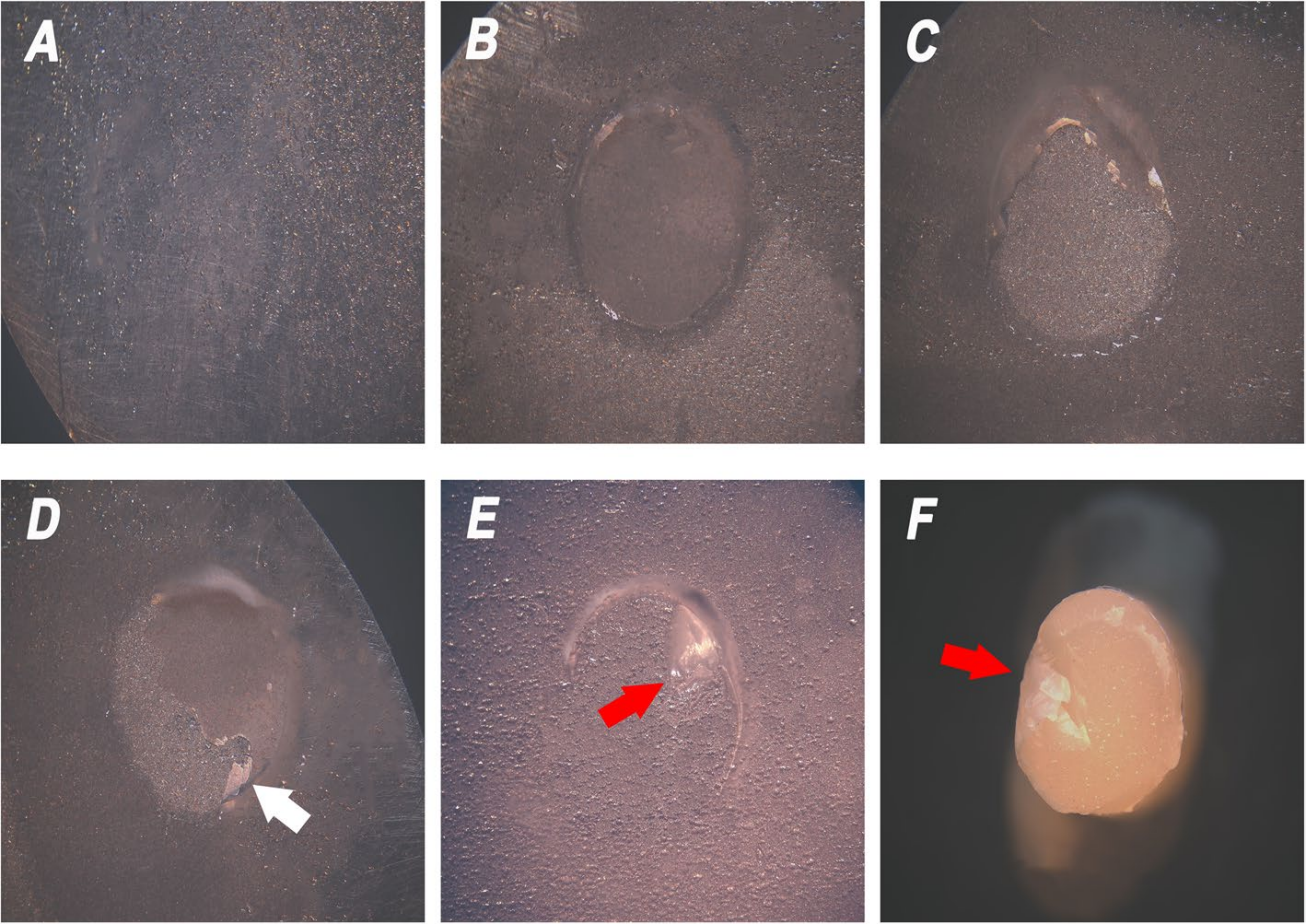
Stereomicroscope images (× 35) of mode of failure after SBS: (**A**) Type III failure (adhesive between resin cement and titanium), (**B**) Type IV failure (adhesive between resin cement and Varseosmile resin), (**C**) Type V mixed failure (Types III and IV combined), (**D**) Type V mixed failure (Types I, III and IV combined) and (**E**, **F**) Type V mixed failure (Types II, III and IV combined). White arrow pointing at cohesive failure in resin cement. Red arrow pointing at cohesive failure in VS resin

**Fig. 4:**
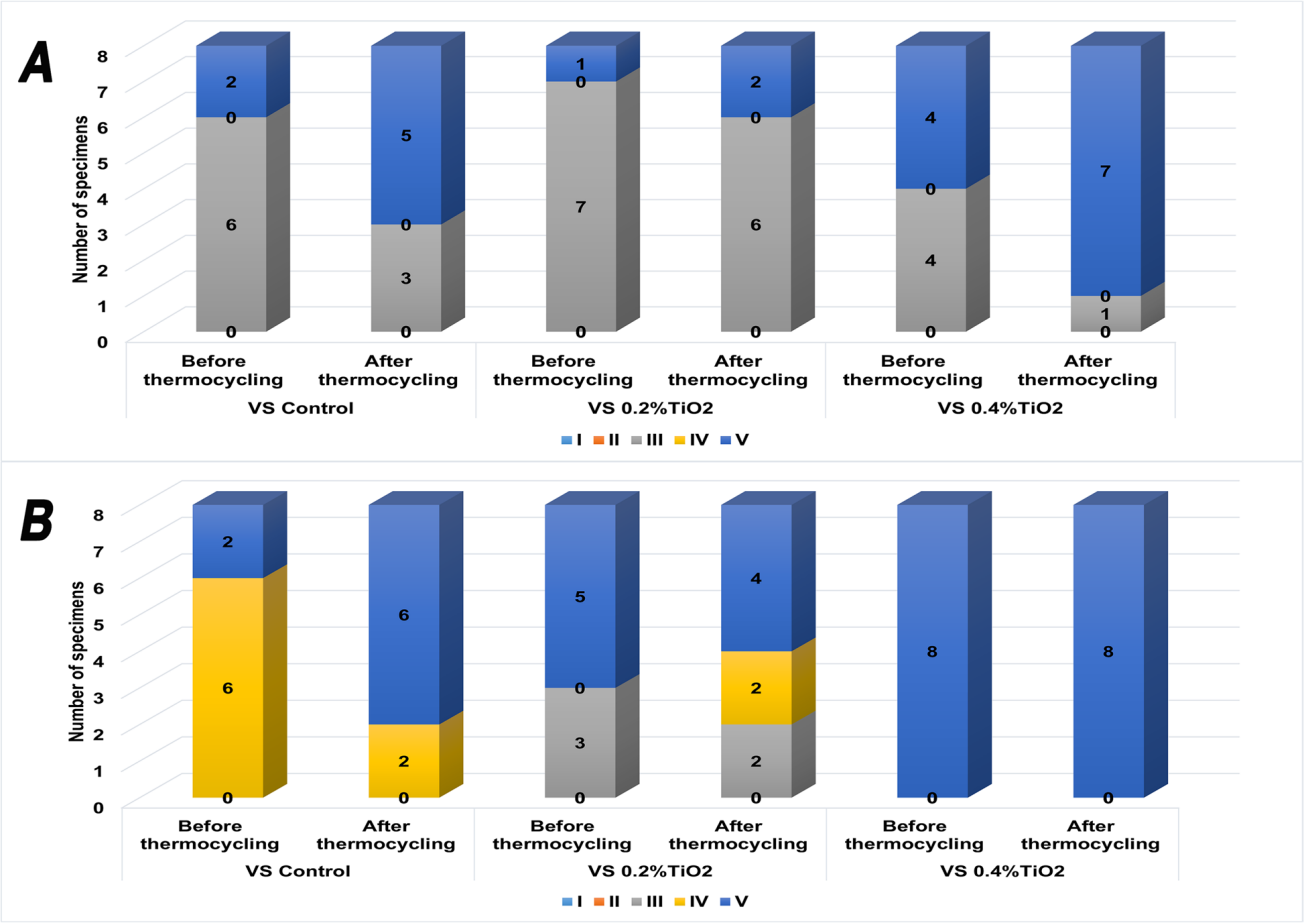
Mode of failure distribution among the specimens after SBS before and after thermocycling on (**A**) smooth titanium surfaces and (**B**) rough titanium surfaces

Figure 3 shows the stereomicroscope images of the modes of failure obtained in this study. Types I and II failure modes were not seen in any of the specimens. As per Fig. 4, the predominant mode of failure on smooth titanium surfaces was type III, which is the adhesive failure between resin cement and the titanium surface, while on rough surfaces type V (mixed failure) prevailed. Type IV appeared only on rough titanium surfaces bonded to VS control and VS 0.2% TiO_2_ groups.

## Discussion

This study discussed the effect of surface treatment by sandblasting and citric acid etching on SBS between titanium dioxide-filled 3D printed photocurable resin and smooth or rough titanium surfaces. The null hypothesis was rejected because there was a significant difference in SBS of 3D printed resin modified with titanium dioxide nanoparticles when bonded to smooth titanium surfaces or sandblasted ones.

The results show that the roughness of titanium surfaces increased significantly after sandblasting (Table 1). This is consistent with a study by Kemarly et al., who proved that sandblasting not only roughens the surface but also removes contaminants, creating a cleaner bonding interface [13]. The resulting rough surface increases the contact area, which promotes better bonding between resin cement and the titanium surface, a feature particularly useful in cement-retained crowns and bridges on titanium abutments [28].

As for the 3D printed resin, the micro-irregularities created by alumina particles contribute to enhanced bond strength of the specimens [29]. Surface irregularities were most prominent in the control group, followed by group II then group III. This trend may be explained by the resistance of titanium dioxide particles to the abrasive effects of alumina particles [30]. Despite this, the surface treatment had a significant contribution to the increased roughness of all groups as sandblasting was followed by citric acid etching, leading to improvement in surface roughness (Table 2).

As discussed previously, the rough surfaces created by the sandblasting of the titanium surfaces enhanced the SBS with the resin, which explains the increased SBS values of all groups bonded to rough titanium surfaces before and after thermocycling when compared to smooth titanium (Table 3). These results are in line with the ones reported by Nakhaei et al., where the sandblasted titanium surfaces offered higher SBS values compared to smooth surfaces regardless of the resin cement used [31]. The mean SBS value of the VS control group when bonded to rough titanium surfaces in our study (8.85 ± 1.03) is higher than the mean SBS obtained by Donmez et al. for the same type of 3D printed resin, which was also bonded to rough titanium surfaces using different resin cements (7.18 ± 2.97) [18]. This might be attributed to the effect of citric acid treatment and the type of resin cement used in this study.

Despite the lower irregularities found on the surfaces of the titanium dioxide-filled resin compared to the control group, the SBS of the VS 0.4%TiO_2_ group was almost comparable to that of the VS control. This effect can be attributed to the conditioning effect of citric acid, which served as an adhesion promoter on the roughened surface of the specimens. Such an effect occurred due to a chemical interaction between titanium dioxide and the resin, potentially via hydrogen bonding between the resin's carbonyl groups and the surface hydroxyl groups of titanium dioxide, which were activated after citric acid application [32]. On the contrary, group II had the lowest SBS values of all groups (Table 3) despite the presence of titanium dioxide particles and the conditioning effect of the citric acid, probably due to the minimized effect of citric acid on this group, as the reduced surface irregularities likely limited citric acid retention compared to the control group as well as the lower loading percentage of titanium dioxide particles compared to group III.

The presence of adhesive functional molecules in the resin cement used in our work, such as 4-META and GPDM also had a significant contribution in enhancing the SBS. These molecules have an effect on both titanium and the 3D printed resin. The titanium dioxide, which in our case is present on both the titanium surface and as nanoparticles in the filled resin, bonds to the carboxylic and phosphate groups present in the functional monomers [16]. Although the bond strengths obtained in this study are lower than those obtained by Fouquet et al., who used different types of cements with different primers, they serve the main objective of using this 3D printed resin as an implant-supported cement-retained interim restoration [17].

The observed increase in SBS across all groups following thermocycling may be attributed to the role of citric acid as a crosslinking agent. The applied heat likely facilitated a thermochemical reaction between citric acid and both the nanocomposites and adhesive resin, where ester bonds formed between the carboxyl groups of citric acid and the hydroxyl groups present in the materials [33]. Additionally, a mild dissolution of titanium dioxide by the carboxylic acid groups in citric acid may have resulted in chelate complex formation at the surface, which could explain the enhanced bond strength in group III. This is likely due to the higher particle loading, providing an increased number of titanium dioxide particles [34]. These results contradict most of the studies conducted on resin cements bonded to titanium specimens, as there is a detrimental effect of thermal cycling on the bond interface leading to deterioration of bond strength [35–37].

Mixed failures are commonly associated with higher SBS values, as they suggest a stronger initial bond that nonetheless fails at multiple layers under stress. In contrast, adhesive failures indicate weaker bonding at the interface, which explains the decrease in their prevalence when the 3D printed resin was bonded to rough titanium surfaces (Fig. 4B) [38, 39]. All groups showed an increase in mixed failure mode (Type V) after thermocycling, which asserts the fact that the increase in temperature has promoted the cross-linking effect of citric acid on polymers. This effect was most heightened in group VS 0.4% TiO_2_ where all its specimens showed mixed failures.

Limitations of this study include using only one type of resin and only two concentrations of the titanium dioxide nanoparticles. Additionally, only one size of alumina particles was utilized during the sandblasting process, and only one concentration of citric acid was used.

## Conclusion

Within the limitations of this study, it could be concluded that the combined effect of surface treatment, both mechanical and chemical, coupled with titanium dioxide nanoparticle addition to 3D printed dental resin restorations have a strong potential to improve the longevity of long-term interim implant-supported restorations. The findings highlight the potential of combining nanotechnology with appropriate surface preparation techniques to improve the clinical performance of composite-to-titanium bonds. Future research should explore the long-term stability of these bonds and their behavior under dynamic intraoral conditions to validate their clinical applicability.

## Supplementary Information

Supplementary Material 1: Table 1. Pairwise comparisons among groups regarding shear bond strength (SBS) of 3D printed resin to smooth and rough titanium surfaces.

## Data Availability

All data generated or analyzed during this study are included in this published article.
