# Correction: Evaluation of 3D printed nano-modified resin shear bond strength on titanium surfaces (an in-vitro study)

**DOI:** 10.1186/s12903-025-06629-4

**Published:** 2025-07-19

**Authors:** Noha Sabry ElMalah, Yomna Ibrahim, Dawlat Mostafa

**Affiliations:** 1 Dental Biomaterials, College of Dentistry, The Arab Academy for Science and Technology and Maritime Transport (AASTMT), El-Alamein, Egypt; 2 Dental Biomaterials Department, Faculty of Dentistry, Dental Biomaterials, Alexandria University, Alexandria, Egypt; 3 Dental Biomaterials, College of Dentistry, The Arab Academy for Science and Technology and Maritime Transport (AASTMT), El‑Alamein, Egypt

**Correction to: BMC Oral Health (2025) 25:806**

10.1186/s12903-025-06223-8

In this article [1], the affiliation details for the author Dawlat Mostafa were incorrectly given as “Dental Biomaterials Department, Faculty of Dentistry, Dental Biomaterials, Alexandria University, Alexandria, Egypt” but should have been “Dental Biomaterials, College of Dentistry, The Arab Academy for Science and Technology and Maritime Transport (AASTMT), El-Alamein, Egypt”.
