# Supplementary material for: Evaluation of 3D printed nano-modified resin shear bond strength on titanium surfaces (an in-vitro study)
Source: BMC Oral Health. 2025 May 27;25:806. doi: 10.1186/s12903-025-06223-8 (PMC12107851; doi:10.1186/s12903-025-06223-8)
Supplement: Supplementary file 1 — Supplementary Material 1: Table 1. Pairwise comparisons among groups regarding shear bond strength (SBS) of 3D printed resin to smooth and rough titanium surfaces. [file 12903_2025_6223_MOESM1_ESM.docx]

**Supplemental table 1. Pairwise comparisons among groups regarding shear bond strength (SBS) of 3D printed resin to smooth and rough titanium surfaces**

| Group | Compared with | *P* | | | |
| --- | --- | --- | --- | --- | --- |
|  |  | SBS on smooth titanium surface | | SBS on rough titanium surface | |
|  |  | Before thermocycling | After  thermocycling | Before thermocycling | After  thermocycling |
| Group I | Group II | 0.11 | 1.00 | 0.33 | 0.05 |
|  | Group III | 1.00 | 1.00 | 1.00 | 0.02* |
| Group II | Group III | 0.89 | 0.67 | 1.00 | <0.0001* |

*Statistically significant difference (*P*<0.05)
